# Novel Derivatives of Nitrobenzofurazan with Chromogenic and Fluorogenic Properties

**DOI:** 10.3390/molecules28166146

**Published:** 2023-08-20

**Authors:** Alexandru Bujor, Anamaria Hanganu, Rodica Baratoiu, Elena N. Hristea, Madalina Tudose, Victorita Tecuceanu, Augustin M. Madalan, Petre Ionita

**Affiliations:** 1 Department of Inorganic, Organic Chemistry, Biochemistry and Catalysis, Faculty of Chemistry, University of Bucharest, 90 Panduri, 050663 Bucharest, Romania; alexandru.bujor@drd.unibuc.ro (A.B.); anamaria_hanganu@yahoo.com (A.H.); augustin.madalan@chimie.unibuc.ro (A.M.M.); 2 Institute of Organic and Supramolecular Chemistry, Spl. Independentei 202B, 060023 Bucharest, Romania; vichi_tecu@yahoo.com; 3 Institute of Physical Chemistry, Spl. Independentei 202, 060023 Bucharest, Romania; rodicad2003@yahoo.com (R.B.); enhristea04@gmail.com (E.N.H.); madalina_tudose2000@yahoo.com (M.T.)

**Keywords:** NBD, fluorescence, synthesis

## Abstract

Five new derivatives were obtained utilizing 4-chloro-7-nitrobenzofurazan (NBD-chloride) in combination with furfurylamine, adamantylamine, aminohippuric acid, phenylalanine, and dehydroabietylamine. These derivatives were then subjected to a comparative analysis of their physical, chemical, and certain biological properties alongside two analogous and known compounds derived from the glycine and 4-amino-TEMPO free radical.

## 1. Introduction

4-Chloro-7-nitro-2,1,3-benzoxadiazole, also known as 4-chloro-7-nitrobenzofurazan or NBD-chloride (NBD-Cl), is a versatile chemical compound that easily converts amines into corresponding fluorescent derivatives through a rapid and highly yielding nucleophilic substitution reaction. NBD-Cl, a non-fluorescent fluorophore, finds wide applications in biology and biomedicine as a fluorescent marker or label for various biomolecules, including peptides and proteins. NBD derivatives of biological thiols, such as cysteine, hold significant importance as well [1,2,3,4,5,6].

The NBD moiety was first encountered in 1968 [7] as a very useful fluorescent label with broad applications in chemistry and biology, yielding highly fluorescent derivatives, although their reactions may not always proceed smoothly. NBD derivatives are also known for their biological activities, such as anti-arthritic and anti-oxidative behaviour [8], and their applications in medicine [9], among others.

Apart from their colour range, which varies from yellow to red, it is worth emphasizing that the most crucial characteristic of NBD-amino derivatives is their extremely intense fluorescence. Thus, NBD-Cl is employed for its chromogenic and fluorogenic properties as a derivatizing agent in analytical chemistry [10,11]. Consequently, colourless and non-fluorescent compounds containing primary or secondary amino groups, such as amines, amino acids, and amino sugars, can be converted into NBD derivatives, enabling their detection via fluorescence or UV-Vis spectroscopy [12]. Furthermore, NBD-Cl serves as a reagent for the synthesis of other valuable benzo-oxadiazole-type compounds used in various physical, chemical, or biological processes [13]. For instance, medicines like cephalosporins [14], acetylcysteine, captopril [15], serotonin [16], glucosinolates [17], ciprofloxacin [18], or materials like mesoporous silica [19] or polymer microparticles [20] have been derivatized with NBD-chloride.

Given the propensity of NBD chloride to readily react with amines, our study focused on a selection of primary amines possessing significant biological or other important properties. These amines include glycine, 4-amino-TEMPO free radical (4-amino-2,2,6,6-tetramethylpiperidine-1-oxyl free radical), furfurylamine, adamantylamine, aminohippuric acid, phenylalanine, and dehydroabietylamine. Glycine, phenylalanine, and aminohippuric acid are natural compounds belonging to the amino acid group. The 4-amino-TEMPO free radical is a stable paramagnetic compound that is utilized in the spin-labeling of other biological substances [21]. Furfurylamine and adamantylamine serve as precursors for drugs, while dehydroabietylamine is classified as a diterpene.

## 2. Results and Discussion

### 2.1. Synthesis

**Figure 1:**
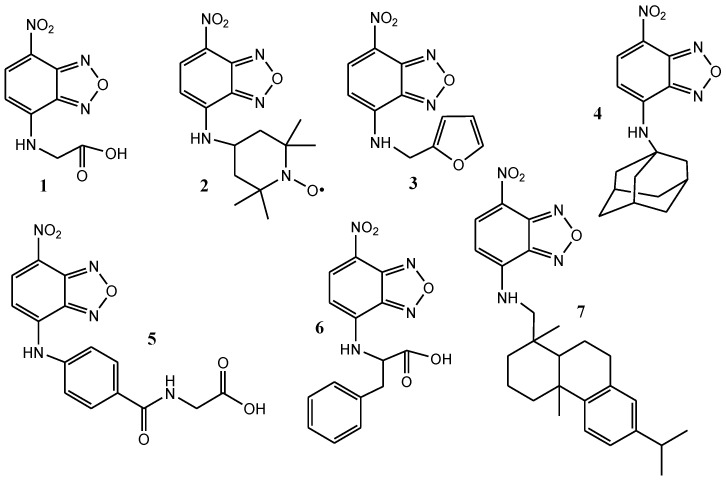
Chemical structure of compounds **1**–**7**.

The corresponding derivatives **1**–**7** (compounds **1** and **2** are known in the literature [22,23], while compounds **3**–**7** are novel) were obtained via the one-step reaction of NBD-chloride with the respective amines in the presence of a base. Figure 1 illustrates these compounds. Several tests were conducted to determine the optimal working conditions for achieving a smooth synthesis and easy separation of the desired compound from the reaction mixture. These tests also accounted for variations in solubility and reaction rates, leading to the following conclusions: compounds **1** and **6**, derived from the amino acids glycine and phenylalanine, respectively, exhibited the best results when a mixture of methanol and water were used as the solvent, along with sodium hydrogen carbonate as the base; compounds **2** and **3** were obtained in acetonitrile with sodium hydrogen carbonate as the base; compounds **4** and **7** were synthesized in dichloromethane (DCM) using triethylamine as the base; lastly, compound **5** was obtained in a mixture of water and acetonitrile with sodium hydrogen carbonate as the base. Subsequently, pure compounds were obtained through column chromatography. The yields of these reactions ranged between 25 and 85%, with the lowest yield observed for compound **4** and the highest for compound **3** (refer to the Experimental section for more details).

### 2.2. Crystal Structure of Compound **3**

**Figure 2:**
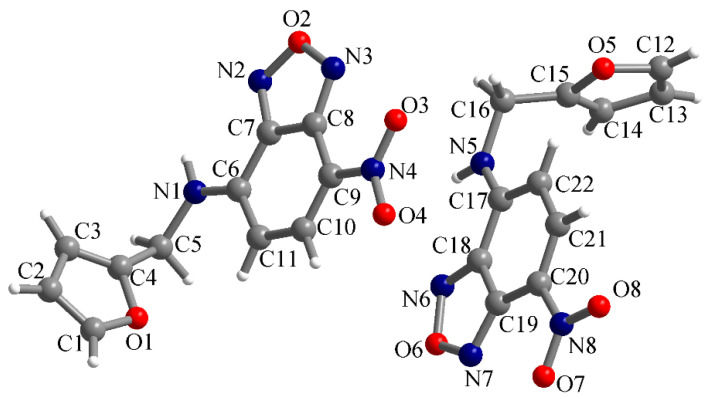
View of the asymmetric unit in the crystal structure of compound **3**.

The slow evaporation of the solution of **3** in DCM led to yellow-red crystals that were suitable for X-ray analysis; therefore, the molecular structure of compound **3** was reconfirmed by X-ray diffraction on a single crystal. Compound **3** crystallized in the orthorhombic *Pca21* space group with two crystallographically independent molecules in the asymmetric unit (Figure 2). In both molecules, the nitro groups were practically coplanar with the benzene ring. The dihedral angles between the mean planes of the nitro groups and their corresponding benzene rings were measured to be 4.3° (for the O3-N4-O4 nitro group) and 3.8° (for the O7-N8-O8 nitro group). The bond lengths involving heteroatoms are provided in Table 1.

**Figure 3:**
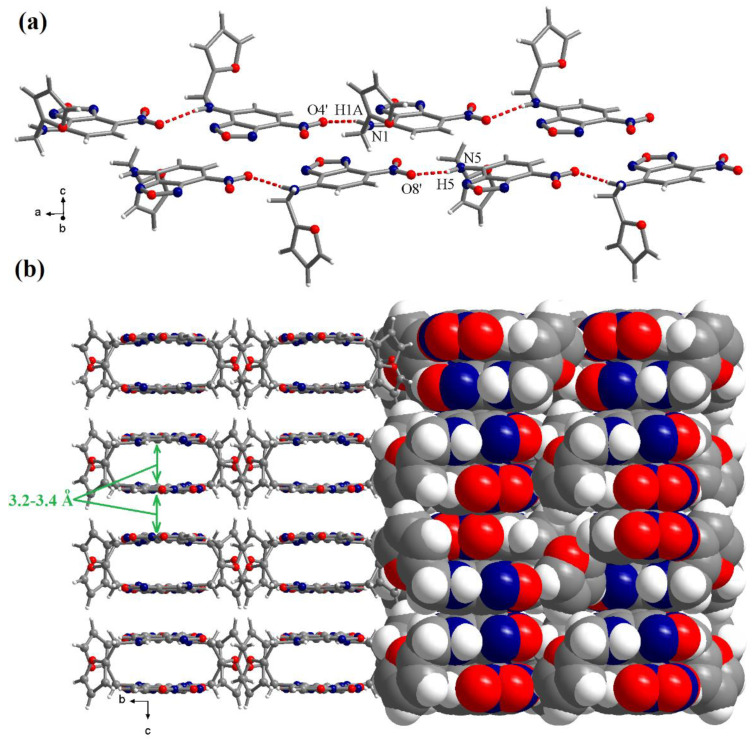
Views of packing diagrams showing the supramolecular chain formed by hydrogen interactions (**a**) and the π–π stacking—view along the crystallographic *a* axis (**b**).

Secondary amino groups are involved in hydrogen interactions with oxygen atoms from nitro groups belonging to neighbouring molecules. Hydrogen interactions generate supramolecular chains which run along the crystallographic *a* axis (Figure 3a). The distance for the hydrogen interactions are: (N1)H1A···O4′ = 2.204 Å and (N5)H5···O8′ = 2.197 Å. The corresponding angles are: N1-H1A···O4′ = 162.6° and N5-H5···O8′ = 167.6° (symmetry code: ’ = 0.5 + x, 1 − y, z). The benzene- and oxadiazole-fused rings, together with nitro groups from neighbouring chains, are placed in a parallel manner (in the crystallographic *ab* planes), generating flat tubes running along the crystallographic *a* axis (Figure 3b). The sides of the tubes are formed by the furan rings. The cavities within the tubes are due to the close p–p interactions between the aromatic systems within the tube (3.2–3.4 Å). Similar distances are also observed for π–π interactions between the supramolecular tubes.

### 2.3. Structural Characterisation via UV-Vis, Fluorescence, IR, and NMR

**Figure 4:**
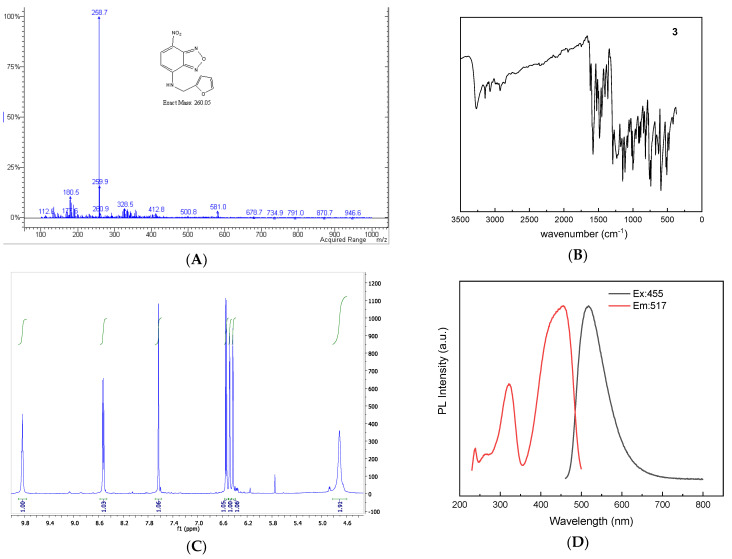
MS (**A**), IR (**B**), ^1^H-NMR (**C**), UV-Vis and fluorescence (**D**) spectrum of compound **3**.

As mentioned previously, NBD derivatives are highly coloured compounds, exhibiting a bright yellow colour in the solution and a red-brown colour in their solid form. The UV-Vis spectra of compounds **1**–**7** mainly display two absorption bands. The first band falls within the range of 312–390 nm, while the second band is observed between 430 and 480 nm (refer to Table 2 for precise values). The first band primarily corresponds to the nitro group, which typically absorbs at lower wavelengths (around 330 nm), while the second band is attributed to the benzofurazan moiety at around 450 nm. Compound **3**, for which we were able to obtain monocrystals, was selected as the representative derivative. Figure 4 illustrates its main spectral characteristics, including UV-Vis, fluorescence, IR, and 1H-NMR spectra. Additionally, selected spectra for the other compounds can be found in the Supplementary Material, and the corresponding data values are provided in the Experimental section. The Stokes shifts (difference between the absorption and emission bands) typically fell within the range of 60–90 nm (refer to Table 1).

The IR spectra reveal several prominent stretch bands. Similar to the previous case, compounds **1**–**7** exhibited strong absorption bands in the regions of 1300–1500 cm^−1^ and 1500–1600 cm^−1^, which could be attributed to the stretching vibrations of the nitro group and the nitrobenzofurazan moiety, respectively. The NH group appeared around 3300–3500 cm^−1^, and aliphatic CH stretching was observed in the range of 2900–3000 cm^−1^, while aromatic CH stretching occurred at approximately 3000–3100 cm^−1^. These observations further confirm the structures of compounds **1**–**7**.

NMR data provide comprehensive details about the structure of compounds **1**–**7** (except **2**, which is a stable free radical, for which the ESR spectrum was recorded, showing the expected triplet with the *a_N_* value around 14 Gauss, see Supplementary Material Figure S2A, while for the ESI-MS spectrum see Supplementary Material Figure S2B). Thus, ^1^H- and ^13^C-NMR spectra confirmed the proposed structures, as the main features of the NBD moiety were represented by the two doublets that were found around *d* values at 8.5 ppm and 6.2 ppm, respectively; all other shifts were well-fitted to the molecular structure (see Experimental for details). Moreover, the MS spectra were recorded and again confirmed the molecular structure of the compounds (see Experimental and Supplementary Material for details).

### 2.4. Lipophilicity, Total Antioxidant Capacity (TAC), and Theoretical Calculations

The significance of lipophilicity in determining the potential candidacy of a molecule as a drug is widely recognized. Lipophilicity, also referred to as hydrophobicity, plays a crucial role in aspects such as metabolism, toxicity, bioavailability, and membrane permeability. The previous literature has demonstrated the use of NBD-derivatized fatty amines to study drug permeation through biomembranes [24,25]. In our study, we functionalized a diterpene amine, dehydroabietylamine (also known as leelamine), with the NBD moiety.

The partition coefficient, often represented as log *p*, can be measured experimentally using methods like biphasic partitioning between *n*-octanol and water, reverse-phase thin-layer chromatography (RP-TLC), or high-performance liquid chromatography (HPLC). Alternatively, it can be calculated theoretically using dedicated software programs. To gain a comprehensive understanding and accurate evaluation of lipophilicity (which is synonymous with hydrophobicity), we employed two methods in our measurements. The first was an experimental approach using RP-TLC, which allowed us to measure the lipophilicity index (*R_M_*_0_). The second was a theoretical (computational) method [26], which provided log *p* values. These measurements were chosen for their simplicity and rapidity. In the experimental RP-TLC method, a non-polar stationary phase (C18 derivatized silica gel) was utilized, with a mixture of acetone and water serving as the eluent. This approach enabled the determination of the experimental lipophilicity (*R_M_*_0_) and the specific hydrophobic surface (*b*) using Equations (1) and (2).

(1) $$R_{M}=\log(\frac{1}{R_{F}}-1)$$

(2) $$R_{M}=R_{M_{0}}+bC$$

**Figure 5:**
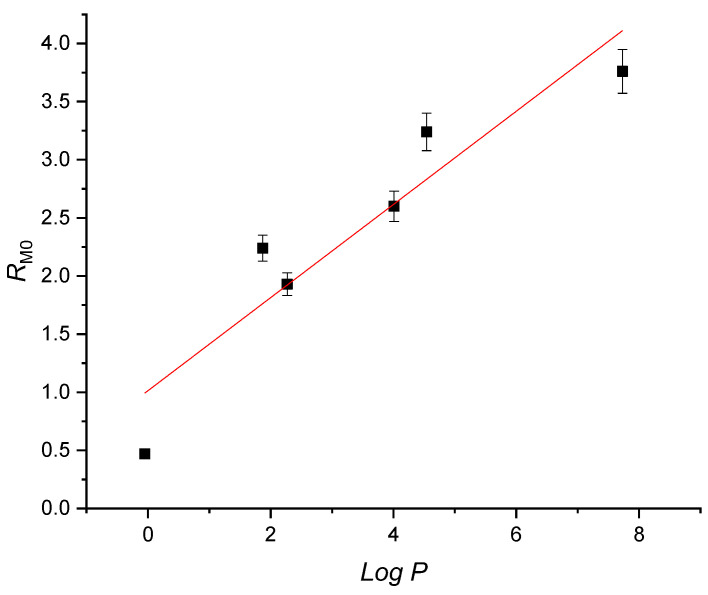
Correlation between *R_M_*_0_ and the partition coefficient log *p* (compound **5** was excluded as peculiar behaviour was encountered in the RP-TLC experiments, see also Table 2).

The obtained values are compiled in Table 2, revealing a noticeable correlation between the experimental lipophilicity (*R_M_*_0_) and the theoretical partition coefficient (log *p*). The lowest values were observed for compound **1**, indicating their higher hydrophilicity due to a smaller hydrocarbon organic moiety and the presence of a carboxylic group, which enhanced their affinity towards aqueous phases. Conversely, the highest values were found for compound **7**, which possessed a significantly larger hydrophobic portion. Table 2 further demonstrates a linear correlation between the experimental lipophilicity (*R_M_*_0_) and the partition coefficient (log *p*) (correlation coefficient r = 0.876), as depicted in Figure 5. Additionally, there was a similar correlation between both theoretical and experimental lipophilicity and the specific hydrophobic surface (b) with the polar surface, as shown in Table 2. With the aim of finding out other possible correlations between the structure and properties, we tried also, besides linear fit, the polynomial fit or multiple linear regression between antioxidant values, log *p*, PSA, absorption or emission wavelength, etc. The correlation coefficient obtained was quite low in most cases (values of correlation coefficient r between 0.01 and 0.34), with the highest values gained for a linear correlation between PSA and *R_M_*_0_ (0.62) and a polynomial fit between PSA, *R_M_*_0,_ and log *p* (0.68). Of course, this demonstrated again the link between PSA, *R_M_*_0,_ and log *p*, as these values represent the balance between the polar and non-polar parts of the molecule.

Total antioxidant capacity (TAC) was measured using the most common method, well-known as the DPPH assay. Thus, the main procedure consists of following the fading of the pink-violet solution of the 2,2-diphenyl-1-picrylhydrazyl stable free radical (DPPH) after the addition of a compound with antioxidant capacity, and therefore, TAC is reported as the percentage of values following Equation (3), where *Abs_i_* refers to the initial absorbance of the mixture, while the *Abs*_30 min_ refers to the absorbance measured after 30 min.

(3) $$TAC\;\left(\%\right)=\frac{\left(Abs\;i-Abs\;30\;\min\right)}{Abs\;i}\times 100$$

Table 2 compiles the values obtained, showing that compound **3** had the highest TAC value (~40%), while the lowest was registered for compound **5** (~8%); these values might be correlated with some structural motifs, taking into account that all derivatives are secondary amines, and in addition, the double bonds and the oxygen atom from the furan ring in compound **3** could provide more easily detected electrons, compared with the saturated adamantan moiety in compound **5**.

PSA values are predictive of drug transport properties, as the sum of the surface areas of polar atoms has been shown to correlate strongly with drug absorption, permeability, and penetration. In our study, the highest PSA value was obtained for compound **1**, while the lowest value was recorded for compound **7** (refer to Table 2) [27].

As mentioned earlier, additional theoretical calculations were conducted using the HyperChem [28] professional package. Computational studies provide an easy means to evaluate structure–property relationships. Before conducting the analysis, the geometry of all compounds **1**–**7** was optimized using the molecular mechanic’s force field MM+ and, subsequently, the semi-empirical PM3 method. The results of this optimisation can be found in Figure S9 of the Supplementary Materials. Furthermore, as we obtained the crystal structure of compound **3** through X-ray diffraction, we were able to assess the accuracy of molecular modelling. The calculated bond lengths for N1-C5 and N1-C6 in compound 3 were found to be 1.480 and 1.396 Å, respectively, while the experimental values were 1.479 and 1.322 Å, respectively (refer to Table 1). In general, these values exhibited good agreement, and the spatial molecular structure appeared similar as well (see Figure S9 in the Supplementary Materials).

## 3. Materials and Methods

### 3.1. Chemicals, Materials and Methods

All chemicals, solvents and materials were purchased from Sigma-Aldrich, Merck or Chimopar, and were used as received.

Lipophilicity was measured experimentally by the RP-TLC method, using 10 × 10 cm RP-TLC plates (silica gel C18, F254), which were spotted with compounds **1**–**7** dissolved in acetone and eluted with different mixtures of acetone-water in chromatographic tank. The procedure was repeated for several mixtures of acetone-water (5/5, 6/4, 7/3, 8/2, *v/v*). After elution, the *R_f_* values were measured either using the direct observation of the spots (all compounds being coloured in yellow) or using a UV-lamp. Thus, the values obtained using Equations (1) and (2) were converted into the experimental lipophilicity (*R_M_*_0_) and specific hydrophobic surface (*b*) and are compiled in Table 1 (together with the *r* correlation coefficient). Log *p* and *PSA* values were obtained using MolInspiration software.

### 3.2. Apparatus

IR spectra were measured using a Bruker Tensor 27 FT-IR spectrometer (ATR). UV-Vis measurements were performed in DCM using a Cary 4000 UV-Vis Agilent Technologies spectrophotometer. Fluorescence measurements were performed in DCM using a lifetime and steady-state spectrometer-FLSP 920, Edinburgh Instruments. Regarding the NMR spectra, these were measured in chloroform-d1 or DMSO-d6 using a Bruker Advance spectrometer operating at 500 MHz for ^1^H and 125 MHz for ^13^C. As usual, the chemical shifts are reported as δ ppm values, and the residual solvent peaks were used as the internal reference. A Varian 310—MS LC/MS/MS triple quadrupole mass spectrometer fitted with an electrospray–ionisation interface (ESI) was used for the MS spectra. The ESR spectrum was recorded on a Jes-FA 100 Jeol Instrument. For the X-ray diffraction measurements for compound **3** employed a Rigaku XtaLAB Synergy-S diffractometer operating with an Mo-K (λ = 0.71073 Å) micro-focus sealed X-ray tube. The structure was solved using direct methods and refined by full-matrix least-squares techniques based on *F*^2^. The non-H atoms were refined with anisotropic displacement parameters. The SHELX-2018 crystallographic software package was used for calculations. The crystallographic data summary and the structure refinement for compound **3** are given in Table 3. CCDC reference number: 2280342.

### 3.3. Total Antioxidant Activity (TAC %)

A fresh stock solution of DPPH in methanol with the concentration of 2 × 10^−5^ M was made, as well as stock solutions of compounds **1**–**7** with a concentration of 0.5 mg/mL. As the antioxidant reference was used for ascorbic acid. Measurements were made after mixing 1.9 mL of the stock solution of DPPH with 0.1 mL of stock solution for each, following the absorbance measuring at 517 nm (the λ_max_ value of DPPH solution) [29].

### 3.4. Synthesis

*Compound* **1**, *2-((7-nitrobenzo[c][1,2,5]oxadiazol-4-yl)amino)acetic acid,* C_8_H_6_N_4_O_5_, M.W. 238. To 1 mmol glycine (75 mg) suspended in 20 mL, water was added with 3 mmol (250 mg) of sodium bicarbonate and 1.1 mmol of NBD-chloride (220 mg) dissolved into 20 mL of methanol. The mixture was stirred until the next day, acidified with aqueous HCl (1 M), and extracted into ethyl acetate. The organic phase was separated, dried over sodium anhydrous sulphate, filtered off, and the solvent was removed under a vacuum. During chromatography on the silica gel column with ethyl acetate, the methanol eluent afforded the pure derivative. Yield 50%. ^1^H-NMR (500 MHz, DMSO-d_6_, δ ppm, J Hz): 8.37 (d, 1H, H_Ar_, 8.9 Hz), 6.37 (d, 1H, H_Ar_, 8.9 Hz), 3.26 (s, 2H, CH_2_) ppm. ^13^C-NMR (125 MHz, DMSO-d_6_, δ ppm): 144.9, 144.8, 138.1, 120.3, 111.5, 103.7, 49.1 ppm. IR (cm^−1^): 3335, 3231, 2918, 1639, 1553, 1493, 1421, 1285, 1132, 993, 906, 847, 596, 592.

*Compound* **2**, *2,2,6,6-tetramethyl-4-(7-nitrobenzo[c][1,2,5]oxadiazol-4-ylamino)piperidin-1-yl,* C_15_H_20_N_5_O_4_, M.W. 334. To 1 mmol, 4-amino-TEMPO (171 mg) was dissolved into 20 mL of acetonitrile with added 5 mmol (420 mg) of sodium bicarbonate and 1.1 mmol of NBD-chloride (220 mg) dissolved into 10 mL of acetonitrile. The mixture was stirred until the next day, acidified with aqueous HCl (1 M), and extracted into DCM. The organic phase was separated, dried over sodium anhydrous sulphate, filtered off, and the solvent was removed under a vacuum. Chromatography on silica gel column with DCM as the eluent afforded the pure derivative. Yield 65%. IR (cm^−1^): 3427, 3337, 3233, 2918, 1639, 1553, 1493, 1421, 1285, 1134, 1032, 995, 907, 849, 660, 596, 463.

*Compound* **3**, *N-(furan-2-ylmethyl)-7-nitrobenzo[c][1,2,5]oxadiazol-4-amine*, C_11_H_8_N_4_O_4_, M.W. 260. To 1 mmol of furfurylamine (100 mg) dissolved into 20 mL of acetonitrile was added to 5 mmol (420 mg) of sodium bicarbonate and 1 mmol of NBD-chloride (200 mg) dissolved into 10 mL of acetonitrile. The mixture was stirred until the next day, acidified with aqueous HCl (1 M), and extracted into DCM. The organic phase was separated, dried over sodium anhydrous sulphate, filtered off, and the solvent was removed under a vacuum. Chromatography on silica gel column with DCM as the eluent afforded the pure derivative. Yield 85%. ^1^H-NMR (500 MHz, DMSO-d_6_, δ ppm, J Hz): 9.83 (t, 1H, NH, 6 Hz), 8.54 (d, 1H, H_Ar_, 8.9 Hz), 7.63 (d, 1H, H_Ar_, 1.0 Hz), 6.54 (d, 1H, H_Ar_, 8.9 Hz), 6.48 (d, 1H, H_Ar_, 3.0 Hz), 6.43 (m, 1H, H_Ar_) ppm. ^13^C-NMR (125 MHz, DMSO-d_6_, δ ppm): 150.1, 149.8, 144.5, 144.1, 143.0, 137.6, 121.6, 110.6, 108.6, 99.8, 39.5 ppm. IR (cm^−1^): 3271, 1618, 1578, 1526, 1502, 1450, 1364, 1292, 1219, 1177, 1076, 991, 916, 903, 845, 808, 779, 758.

*Compound* **4**, *N-((3s,5s,7s)-adamantan-1-yl)-7-nitrobenzo[c][1,2,5]oxadiazol-4-amine,* C_16_H_18_N_4_O_3_, M.W. 314. To 1 mmol of 1-aminoadamantane (151 mg) dissolved into 20 mL of DCM was added 0.2 mL of triethylamine and 1 mmol of NBD-chloride (200 mg). The mixture was stirred until the next day, acidified with aqueous HCl (1 M), and extracted into DCM. The organic phase was separated, dried over sodium anhydrous sulphate, filtered off, and the solvent was removed under a vacuum. Chromatography on silica gel column with the eluent afforded the pure derivative. Yield 25%. ^1^H-NMR (300 MHz, CDCl_3_, δ ppm, J Hz): 8.42 (d, 1H, H_Ar_, 9.1 Hz), 6.13 (d, 1H, H_Ar_, 9.1 Hz), 2.36–1.06 (m, 15H, H_aliphatic_) ppm. ^13^C-NMR (75 MHz, CDCl_3_, δ ppm): 145.0, 144.6, 144.3, 135.5, 121.7, 100.6, 44.0, 43.1, 35.7, 29.5 ppm. IR (cm^−1^): 3024, 2920, 2851, 1601, 1493, 1450, 1300, 748, 696, 536.

*Compound* **5**, *2-(4-((7-nitrobenzo[c][1,2,5]oxadiazol-4-yl)amino)benzamido)acetic acid,* C_15_H_11_N_5_O_6_, M.W. 357. To 1 mmol 4-aminohippuric acid (194 mg) suspended in a mixture of 5 mL water, 20 mL acetonitrile was added with 3 mmol (250 mg) of sodium bicarbonate and 1.1 mmol of NBD-chloride (220 mg) dissolved into 10 mL of acetonitrile. The mixture was stirred until the next day, acidified with aqueous HCl (1 M), and extracted into DCM. The organic phase was separated, dried over sodium anhydrous sulphate, filtered off, and the solvent was removed under a vacuum. During chromatography on the silica gel column with the ethyl acetate, the methanol eluent afforded the pure derivative. Yield 30%. ^1^H-NMR (500 MHz, DMSO-d_6_, δ ppm, J Hz): 12.05 (s, 1H, NH), 8.26 (t, 1H, NH, 5.5 Hz), 7.87 (d, 2H, H_Ar_, 8.5 Hz), 7.78 (d, 1H, H_Ar_, 10.1 Hz), 6.96 (d, 2H, H_Ar_, 8.5 Hz), 5.71 (d, 1H, H_Ar_, 10.1 Hz), 3.79 (d, 2H, CH_2_, 5.5 Hz) ppm. ^13^C-NMR (125 MHz, DMSO-d_6_, δ ppm):173.5, 165.6, 154.2, 150.3, 148.8, 146.7, 133.3, 128.7, 128.4, 120.4, 110.9, 103.3, 43.1 ppm. IR (cm^−1^): 1904, 1686, 1655, 1626, 1545, 1499, 1441, 1414, 1366, 1288, 1167, 1096, 1051, 1038, 1020, 997, 897, 663, 598, 455. 

*Compound* **6**, *2-(7-nitrobenzo[c][1,2,5]oxadiazol-4-ylamino)-3-phenylpropanoic acid,* C_15_H_12_N_4_O_5_, M.W. 328. To 1 mmol of phenylalanine (165 mg) suspended into 20 mL of water, 3 mmol (250 mg) of sodium bicarbonate and 1.1 mmol of NBD-chloride (220 mg) were added and dissolved into 20 mL of methanol. The mixture was stirred until the next day, acidified with aqueous HCl (1 M), and extracted in DCM. The organic phase was separated, dried over sodium anhydrous sulphate, filtered off, and the solvent was removed under a vacuum. Chromatography on silica gel column with ethyl acetate as the eluent afforded the pure derivative. Yield 65%. ^1^H-NMR (300 MHz, DMSO-d_6_, δ ppm, J Hz): 8.37 (d, 1H, H_Ar_, 8.9 Hz), 7.30 (d, 2H, H_Ar_, 7.2 Hz), 7.18 (t, 2H, H_Ar_, 7.2 Hz), 7.10 (t, 1H, H_Ar_, 7.2 Hz), 6.31 (m, 1H, H_Ar_), 4.70 (m, 1H, CH), 3.39–3.28 (m, 2H, CH_2_) ppm. ^13^C-NMR (75 MHz, DMSO-d_6_, δ ppm): 171.9, 145.0, 144.5, 144.0, 137.7, 136.9, 129.2, 128.2, 126.5, 121.5, 100.0, 58.6, 36.4 ppm. IR (cm^−1^): 3306, 2953, 2916, 2849, 2114, 1709, 1570, 1448, 1296.

*Compound* **7**, *N-((7-isopropyl-1,4a-dimethyl-1,2,3,4,4a,9,10,10a-octahydrophenanthren-1-yl)methyl)-7-nitrobenzo[c][1,2,5]oxadiazol-4-amine*, M.W. 448. To 1 mmol dehydroabietylamine (285 mg) suspended into 50 mL of DCM, 2.5 mmol (0.35 mL) of triethylamine and 1.1 mmol of NBD-chloride (220 mg) was added and dissolved into 50 mL of DCM. The mixture was stirred until the next day, acidified with aqueous HCl (1 M), and extracted into DCM. The organic phase was separated, dried over sodium anhydrous sulphate, filtered off, and the solvent was removed under a vacuum. Chromatography on silica gel column with DCM as the eluent afforded the pure derivative. Yield 55%. ^1^H-NMR (500 MHz, CDCl_3_, δ ppm, J Hz): 8.48 (d, 1H, H_Ar_, 8.7 Hz), 7.18 (d, 1H, H_Ar_, 8.1 Hz), 7.02 (dd, 1H, H_Ar_, 1.5 Hz, 8.1 Hz), 6.89 (d, 1H, H_Ar_, 1.5 Hz), 6.25 (d, 1H, H_Ar_, 8.7 Hz), 6.21 (t, 1H, NH, 5.7 Hz), 3.39 (d, 2H, CH_2_, 5.7 Hz), 2.89 (dd, 1H, CH, 6.2 Hz, 17.2 Hz), 2.89–2.80 (m, 2H, H_aliphatic_), 2.36–2.34 (m, 1H, CH), 1.91–1.75 (m, 4H, H_aliphatic_), 1.67–1.58 (m, 3H, H_aliphatic_), 1.47–1.40 (m, 2H, H_aliphatic_), 1.27 (s, 1H, CH_3_), 1.22 (d, 6H, CH_3_), 1.12 (s, 1H, CH_3_) ppm. ^13^C-NMR (125 MHz, CDCl_3_, δ ppm): 146.32, 145.91, 144.32, 144.21, 143.70, 133.87, 128.90, 128.09, 126.76, 125.16, 124.08, 98.41, 54.64, 46.00, 38.25, 37.97, 37.49, 36.46, 33.31, 29.91, 25.18, 23.82, 19.23, 18.71, 18.34 ppm. IR (cm^−1^): 2986, 2912, 2839, 2347, 2108, 1560, 1439, 1292, 1248, 598.

## 4. Conclusions

As previously established, NBD chloride is a multipurpose reagent that easily transforms colourless and non-fluorescent primary amines into intensely coloured and fluorescent derivatives, although these characteristics are dependent on the molecular structure of the starting material. Following this procedure, five new NBD-derivatives (compounds **3**–**7**) were obtained and structurally characterized by IR, MS, UV-Vis, fluorescence, ^1^H- and ^13^C-NMR; in addition, for compound **3,** the crystal structure was revealed by X-ray diffraction. The synthetic details and knowledge of the structural characteristics of these novel compounds, combined with the biological significance of the amines employed in this study, are expected to facilitate further advancements across diverse scientific fields.

## Figures and Tables

**Table 1:** Selected bond lengths (Å) for compound **3**.

C1-O1 = 1.357(7)	N2-O2 = 1.361(4)	C12-O5 = 1.367(6)	N6-O6 = 1.367(4)
C4-O1 = 1.355(4)	N3-O2 = 1.384(4)	C15-O5 = 1.367(4)	N7-O6 = 1.378(4)
C5-N1 = 1.479(4)	C9-N4 =1.424(4)	C16-N5 = 1.474(5)	C20-N8 = 1.413(4)
C6-N1 = 1.322(5)	N4-O3 = 1.227(4)	C17-N5 = 1.319(5)	N8-O7 = 1.224(3)
C7-N2 = 1.320(4)	N4-O4 = 1.238(4)	C18-N6 = 1.302(4)	N8-O8 = 1.234(4)
C8-N3 = 1.317(4)		C19-N7 = 1.324(4)	

**Table 2:** R_f_ values, wavelength of UV-Vis absorption (λ_max_) and fluorescence emission (λ_em_), total antioxidant capacity (TAC), experimental (*R_M_*_0_) and theoretical (log *p*) lipophilicity, polar-surface area (*PSA*) for compounds **1**–**7**.

Compound	R_f_ ^i^	λ_max_ ^i^	λ_em_ ^i^	TAC ^ii^	log *p* ^iii^	*PSA* ^iii^	*R_M_*_0_ ^iv^	*b* ^iv^	*r* ^iv^
**1**	0.23	312, 432	450	30.3	−0.05	134.07	0.47	−1.2892	0.934
**2**	0.07	312, 430	516	15.1	4.01	120.24	2.60	−3.6403	0.969
**3**	0.51	320, 442	517	40.1	2.27	109.92	1.93	−3.2031	0.878
**4**	0.71	329, 460	525	12.2	4.54	96.78	3.24	−3.8593	0.936
**5**	0	390, 480	550	7.7	1.28	163.17	-	-	-
**6**	0.49	316, 443	526	18.4	1.87	134.07	2.24	−2.9753	0.958
**7**	0.67	328, 466	526	23.9	7.73	96.78	3.76	−4.0273	0.969

^i^ DCM was used as solvent; ^ii^ DPPH method (%); ^iii^ theoretical values calculated with MolInspiration software; ^iv^ experimental values.

**Table 3:** Crystallographic data, details of data collection and structure refinement parameters for compound **1**.

Compound	3
Chemical formula	C_11_H_8_N_4_O_2_
M (g mol^−1^)	260.21
Temperature, (K)	293(2)
Wavelength, (Å)	0.71073
Crystal system	*Orthorhombic*
Space group	*Pca21*
*a* (Å)	17.3128(8)
*b* (Å)	10.0556(6)
*c* (Å)	12.7006(7)
*α* (º)	90
*β* (º)	90
γ (º)	90
V (Å^3^)	2211.1(2)
Z	8
*D_c_* (g cm^−3^)	1.563
*μ* (mm^−1^)	0.123
*F*(000)	1072
Goodness-of-fit on *F*^2^	1.023
Final *R*_1_, w*R*_2_ [I > 2σ(I)]	0.0399, 0.1087
*R*_1_, w*R*_2_ (all data)	0.0505, 0.1167

## Data Availability

Not applicable.

## Supplementary Materials

The supporting information can be downloaded at: https://www.mdpi.com/article/10.3390/molecules28166146/s1, Figure S1. Fluorescence (A) and IR (B) spectrum of compound **1**; Figure S2. ESR (A) and MS (B) spectrum of compound **2**; Figure S3. ^13^C-NMR spectrum of compound **3**; Figure S4. Fluorescence (A) and MS (B) spectrum of compound **4**; Figure S5. Fluorescence (A) and MS (B) spectrum of compound **5**; Figure S6. Fluorescence (A) and MS (B) spectrum of compound **6**; Figure S7. Fluorescence (A) and MS (B) spectrum of compound **7**; Table S1. R_f_ values obtained by RP-TLC; Figure S8. Colour of an acetone solution of compounds **1**–**7** (up) and their fluorescence at 365 nm (bottom); Figure S9. Semi-empirical PM3 geometry optimisation of compounds **1**–**7**.

## Sample Availability

Samples of compounds **1**–**7** are available from the authors.

## References

[1] Zhu Z., Liu W., Cheng L., Li Z., Xi Z., Yi L. (2015). New NBD-based fluorescent probes for biological thiols. Tetrah. Lett..

[2] Mena Y., Li Z., Zhang J., Tong Z., Xi Z., Qiu X., Yi L. (2015). Rational design and synthesis of fast-response NBD-based fluorescent probes for biothiols. Tetrah. Lett..

[3] Shen Y., Zhang X., Zhang Y., Zhang C., Jin J., Li H., Yao S. (2016). A novel colorimetric/fluorescence dual-channel sensor based on NBD for the rapid and highly sensitive detection of cysteine and homocysteine in living cell. Analytic. Meth..

[4] Biswas S., Pal K., Kumar P., Koner A.L. (2018). A fluorogenic probe for in vitro and in vivo detection of biothiols and vitamin-C with an in-depth mechanistic understanding. Sens. Actuators B.

[5] Yu H., Liu Y., Wang J., Liang Q., Liu H., Xu J., Shao S. (2017). A gold nanocluster-based ratiometric fluorescent probe for cysteine and homocysteine detection in living cells. New J. Chem..

[6] Wang J., Niu L., Huang J., Yan Z., Wang J. (2018). A novel NBD-based fluorescent turn-on probe for the detection of cysteine and homocysteine in living cells. Spectrochim. Acta Part A Molec. Biomolec. Spect..

[7] Ghosh P.B., Whitehouse R.D. (1968). 7-chloro-4-nitrobenzo-2-oxa-1,3-diazole: A new fluorigenic reagent for amino acids and other amines. Biochem. J..

[8] Shah S.U.A., Ashraf N., Soomro Z.H. (2012). The anti-arthritic and anti-oxidative effect of NBD (6-nitro-1,3-benzodioxane) in adjuvant-induced arthritis (AIA) in rats. Inflamm. Res..

[9] Boström J., Hogner A., Llinas A., Wellner E., Plowright A.T. (2012). Oxadiazoles in medicinal chemistry. J. Med. Chem..

[10] Almahri A. (2021). Utility of 4-chloro-7-nitrobenzofurazan for spectrofluorimetric and spectrophotometric determinations of the anti-hirsutismagent (α-difluoromethylornithine) in pharmaceutical cream samples. Luminescence.

[11] Kim H.W., Choi M.G., Park H., Leea J., Chang S.K. (2015). Single molecular multianalyte signaling of sulfide and azide ions by a nitrobenzoxadiazole-based probe. RSC Adv..

[12] Yuan Y., Cao F., Yuan G. (2023). Fluorescent-dye-labeled amino acids for real-time imaging in arabidopsis thaliana. Molecules.

[13] Jung M.E., Dong T.A., Cai X. (2011). Improved synthesis of 4-amino-7-nitrobenz-2,1,3-oxadiazoles using NBD fluoride (NBD-F). Tetrah. Lett..

[14] Abdellatef H.E., Shalaby A.A., Elsaid H.M., Ayad M.M. (2000). Colorimetric and titrimetric methods for determination of some cephalosporins in their pure and dosage forms. Sci. Pharm..

[15] Haggag R., Belal S., Shaalan R. (2008). Derivatization with 4-chloro-7-nitro-2,1,3-benzoxadiazole for the spectrophotometric and differential pulse polarographic determination of acetylcysteine and captopril. Sci. Pharm..

[16] Sarkar P., Harikumar K.G., Rawat S.S., Das S., Chakraborty T.K., Chattopadhyay A. (2021). Environment-sensitive fluorescence of 7-nitrobenz-2-oxa-1,3-diazol-4-yl (nbd)-labeled ligands for serotonin receptors. Molecules.

[17] Kanstrup C., Jimidar C.C., Tomas J., Cutolo G., Crocoll C., Schuler M., Klahn P., Tatibouët A., Nour-Eldin H.H. (2023). artificial fluorescent glucosinolates (f-gsls) are transported by the glucosinolate transporters GTR1/2/3. Int. J. Mol. Sci..

[18] Karpushenkova V.S., Glinskaya L.I., Faletrov Y.V., Bardakova K.N., Piskun Y.A., Kostjuk S.V., Shkumatov V.M. (2022). New photochemical properties of azidoaniline and ciprofloxacin. Chem. Proc..

[19] Tudose M., Culita D.C., Baratoiu-Carpen R.D., Mitran R.-A., Kuncser A., Romanitan C., Popescu R.C., Savu D.I. (2022). Novel antitumor agents based on fluorescent benzofurazan derivatives and mesoporous silica. Int. J. Mol. Sci..

[20] Zhang W., Li Q., Zhang H. (2023). Efficient optosensing of hippuric acid in the undiluted human urine with hydrophilic “turn-on”-type fluorescent hollow molecularly imprinted polymer microparticles. Molecules.

[21] Liu J., Cheng R., Eps N.V., Wang N., Morizumi T., Ou W.L., Klauser P.C., Rozovsky S., Ernst O.P., Wang L. (2020). Genetically encoded quinone methides enabling rapid, site-specific, and photocontrolled protein modification with amine reagents. J. Am. Chem. Soc..

[22] Bognár B., Ősz E., Hideg K., Kálai T. (2006). Synthesis of new double (spin and fluorescence) sensor reagents and labels. J. Heter. Chem..

[23] Maike B., Cola L.D., Armido S. (2011). Site-specific immobilization of proteins at zeolite L crystals by nitroxide exchange reactions. Chem. Comm..

[24] Loura L.M.S. (2012). Lateral distribution of nbd-pc fluorescent lipid analogs in membranes probed by molecular dynamics-assisted analysis of förster resonance energy transfer (fret) and fluorescence quenching. Int. J. Mol. Sci..

[25] Filipe H.A.L., Loura L.M.S., Moreno M.J. (2023). Permeation of a homologous series of nbd-labeled fatty amines through lipid bilayers: A molecular dynamics study. Membranes.

[26] molinspiration.com.

[27] Ertl P., Rohde B., Selzer P. (2000). Fast calculation of molecular polar surface area as a sum of fragment based contributions and its application to the prediction of drug transport properties. J. Med. Chem..

[28] Licenced Version 8 Used on, CD. hypercubeusa.com.

[29] Prior R.L., Wu X., Schaich K. (2010). Standardized methods for the determination of antioxidant capacity and phenolics in foods and dietary supplements. J. Agric. Food Chem..

