# Supplementary material for: Novel Derivatives of Nitrobenzofurazan with Chromogenic and Fluorogenic Properties
Source: Molecules. 2023 Aug 20;28(16):6146. doi: 10.3390/molecules28166146 (PMC10459551; doi:10.3390/molecules28166146)
Supplement: Supplementary file 1 [file molecules-28-06146-s001.zip › molecules-2569100-supplementary.pdf]

Supplementary Material

Novel derivatives of nitrobenzofurazan with chromogenic and fluorogenic properties

Alexandru Bujor, Anamaria Hanganu, Rodica Baratoiu, Elena N. Hristea, Madalina Tudose, Victorita Tecuceanu, Augustin M. Madalan and Petre Ionita

List of Figures and Tables

Figure S1. Fluorescence (A) and IR (B) spectrum of compound **1**

Figure S2. ESR (A) and MS (B) spectrum of compound **2**

Figure S3. ^{13}C-NMR spectrum of compound **3**

Figure S4. Fluorescence (A) and MS (B) spectrum of compound **4**

Figure S5. Fluorescence (A) and MS (B) spectrum of compound **5**

Figure S6. Fluorescence (A) and MS (B) spectrum of compound **6**

Figure S7. Fluorescence (A) and MS (B) spectrum of compound **7**

Table S1. R_f values obtained by RP-TLC

Figure S8. Colour of an acetone solution of compounds **1-7** (up) and their fluorescence at 365 nm (bottom).

Figure S9. Semi-empirical PM3 geometry optimization of compounds **1-7**.

Figure S1. Fluorescence (A) and IR (B) spectrum of compound **1**

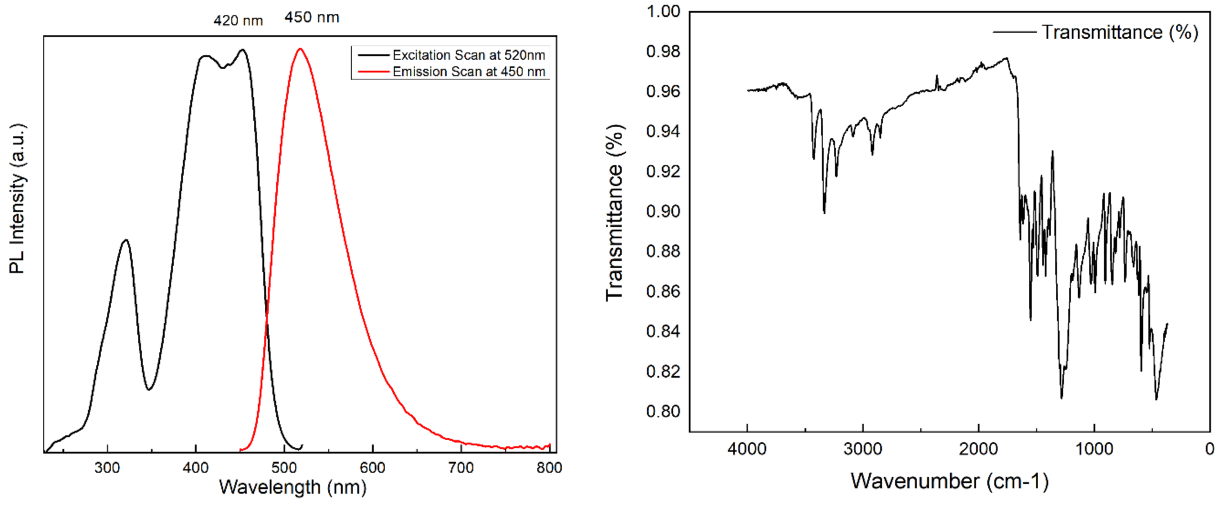

A **B**

Figure S2. ESR (A) and MS (B) spectrum of compound **2**

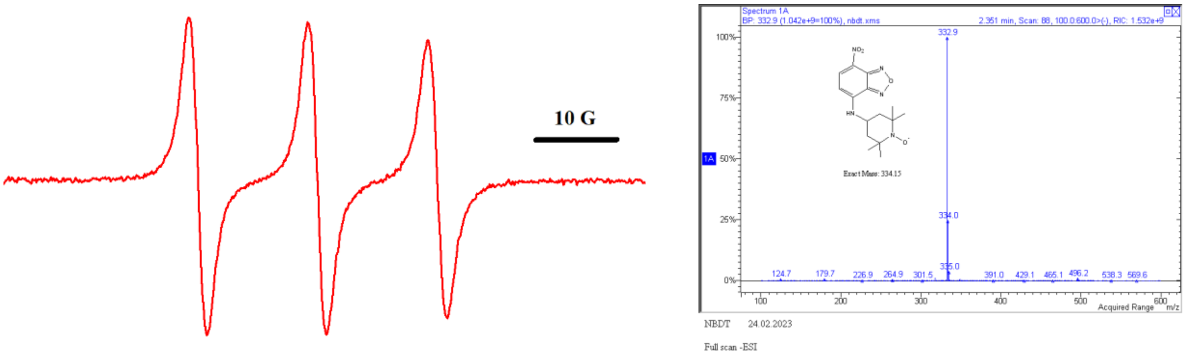

A **B**

Figure S3. ^{13}C-NMR spectrum of compound **3**

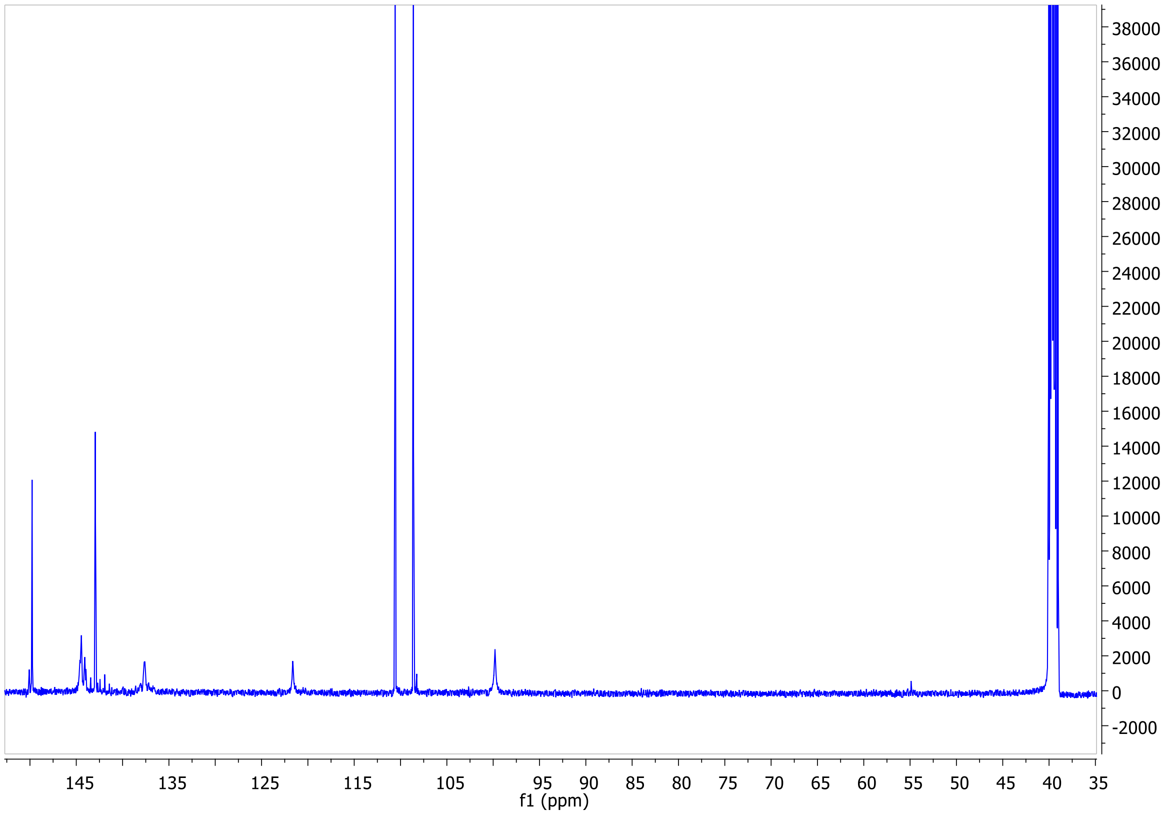

Figure S4. Fluorescence (A) and MS (B) spectrum of compound **4**

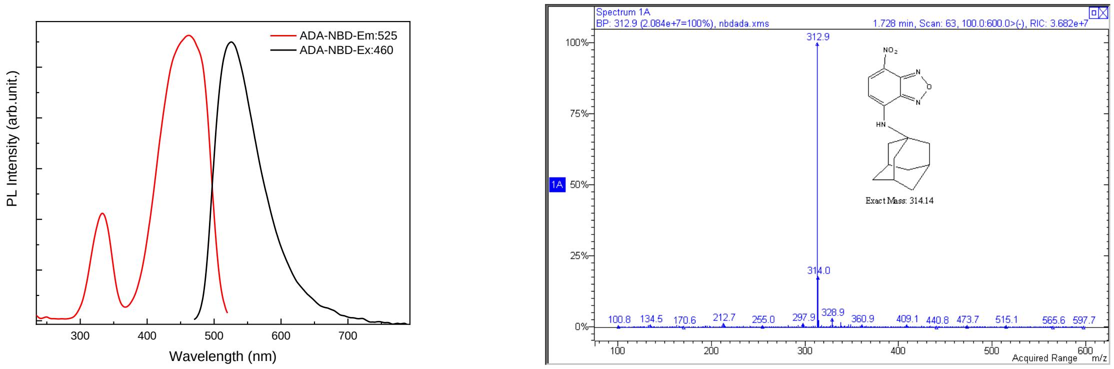

A **B**

Figure S5. Fluorescence (A) and MS (B) spectrum of compound **5**

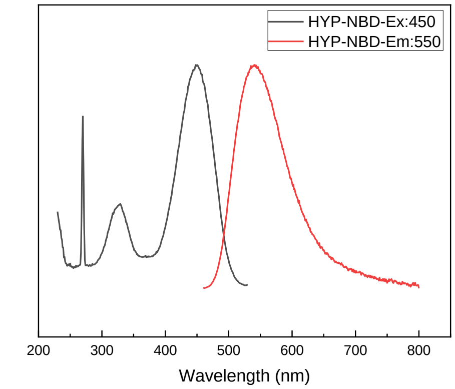

A

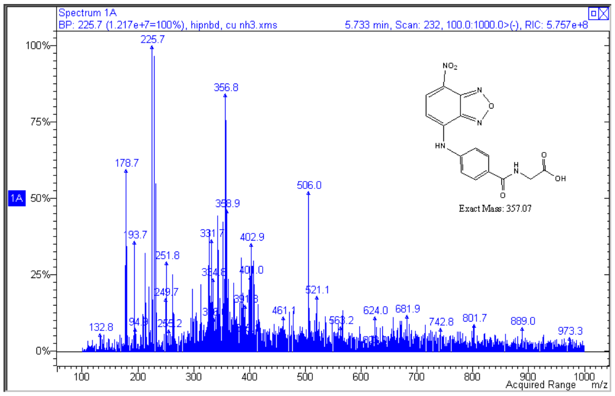

B

Figure S6. Fluorescence (A) and MS (B) spectrum of compound **6**

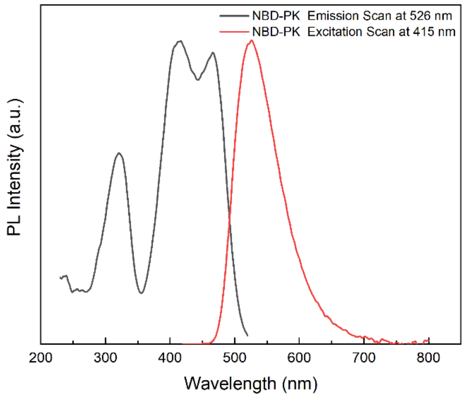

A

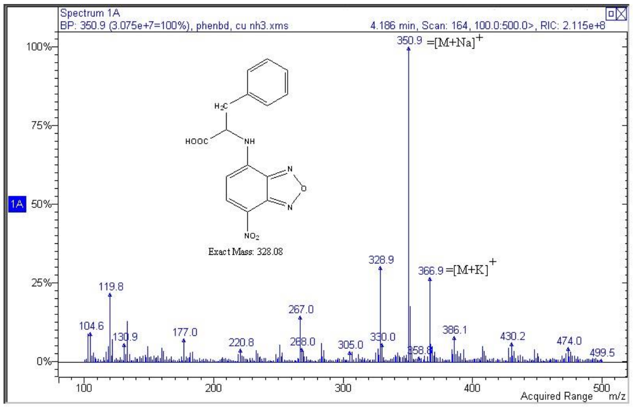

B

Figure S7. Fluorescence (A) and MS (B) spectrum of compound **7**

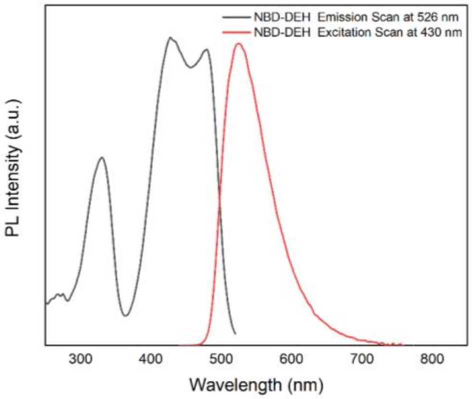

A

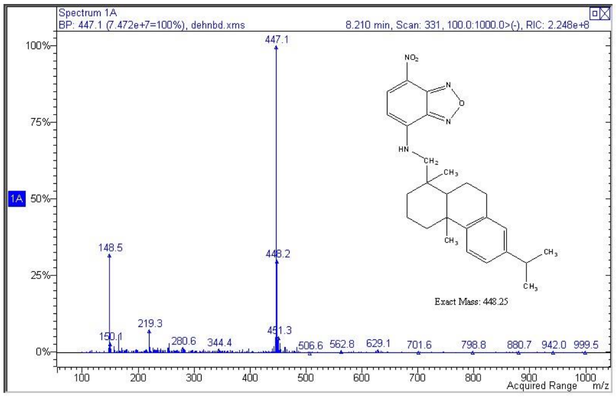

B

Table S1. R_f values obtained by RP-TLC

C/Cmp.	1	2	3	4	5	6	7
0.5	0.62	0.12	0.25	0.06	-	0.17	0.02
0.6	0.63	0.33	0.58	0.08	-	0.21	0.04
0.7	0.73	0.46	0.72	0.19	-	0.42	0.08
0.8	0.79	0.65	0.76	0.47	-	0.59	0.26

Figure S8. Colour of an acetone solution of compounds **1-7** (up) and their fluorescence at 365 nm (bottom).

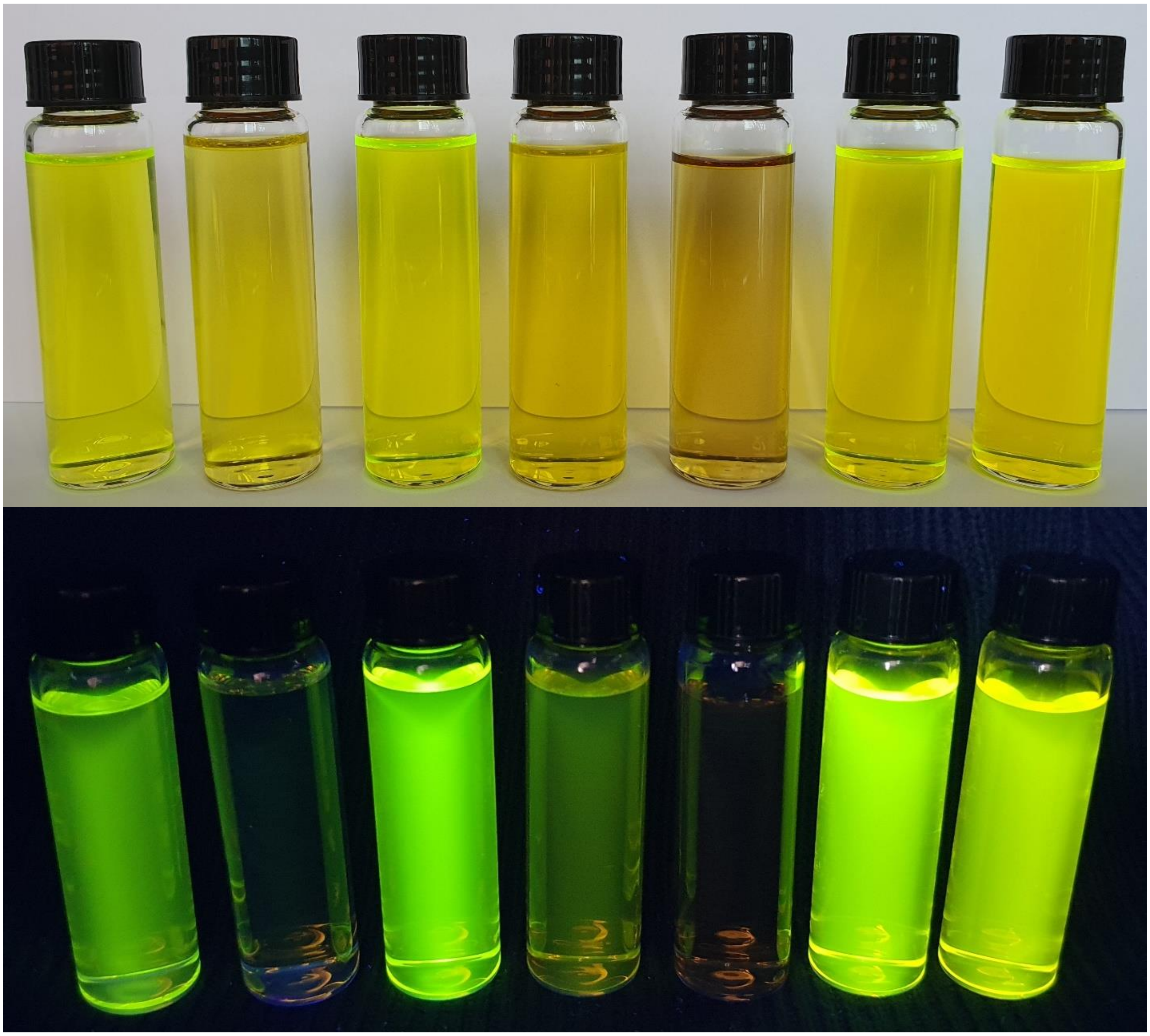

Figure S9. Semi-empirical PM3 geometry optimization of compounds **1-7**.

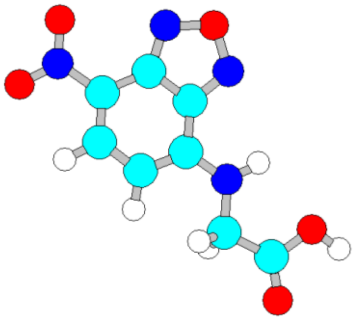

1

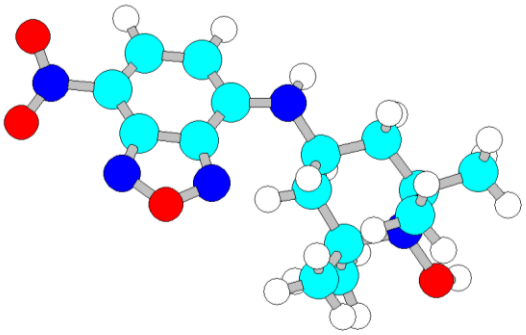

2

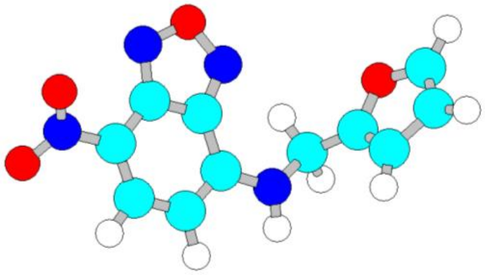

3

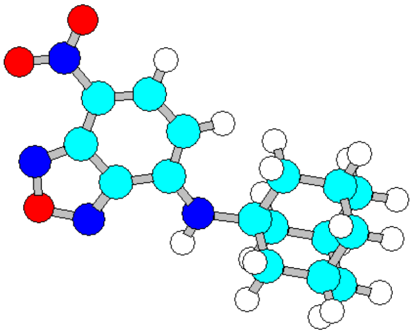

4

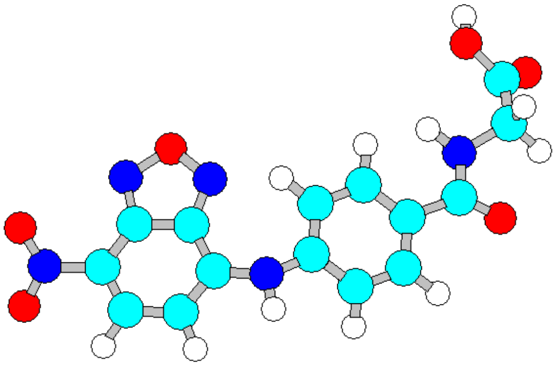

5

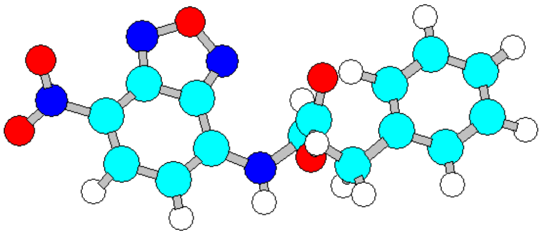

6

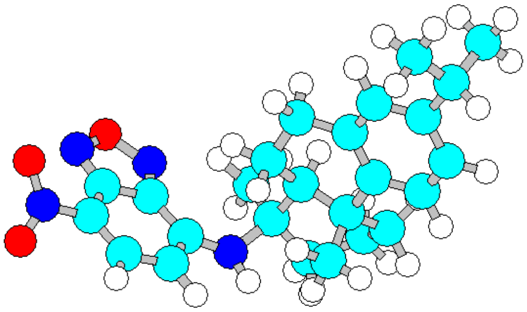

7
